# Associations between childhood adversity and depressive symptoms in later life: parallel mediation through self-rated health, internet media use, and social adaptation among Chinese older adults

**DOI:** 10.3389/fpubh.2026.1941289

**Published:** 2026-10-08

**Authors:** Zhangbo An

**Affiliations:** School of Government, Beijing Normal University, Beijing, China

**Keywords:** childhood adversity, depressive symptoms, internet media use, self-rated health, social adaptation

## Abstract

**Introduction:**

Late-life depressive symptoms remain a major public health concern in aging China. Although childhood adversity is a recognized risk factor for later-life depression, the concurrent conditions that statistically account for the association between early disadvantage and current emotional distress, particularly those amenable to intervention, have not been systematically examined in a parallel mediation framework.

**Methods:**

Using cross-sectional data from the 2023 wave of the China Longitudinal Aging Social Survey (N = 11,670), we examined associations between childhood adversity and depressive symptoms and tested whether self-rated health, internet media use, and social adaptation accounted for part of that association as parallel mediators. Childhood adversity was a composite of childhood hunger, childhood medical deprivation, and parental loss by age 10. Depressive symptoms were assessed with a nine-item Center for Epidemiologic Studies Depression Scale. Models adjusted for sociodemographic covariates and used survey weights.

**Results:**

Childhood adversity was positively associated with depressive symptoms in the total-effect model (*β* = 0.439, *p* < 0.001; *N* = 10,476). In the complete-mediator sample (*N* = 10,093), the corresponding total association was *β* = 0.348. The three concurrent measures together accounted for about 92% of this latter association in the specified model. The largest specific indirect associations were through social adaptation (*β* = 0.147) and self-rated health (*β* = 0.125), followed by internet media use (*β* = 0.051). A bootstrap contrast did not show a statistically significant difference between the social-adaptation and health indirect associations. The remaining direct association was small and nonsignificant (*β* = 0.026, *p* = 0.464). Subgroup analyses showed marked heterogeneity by community activity participation, residence, and family structure.

**Discussion:**

Because the data are cross-sectional, these results describe association decomposition rather than causal transmission. Adversity-linked depressive burden in later life is concentrated in present-day health, social adaptation, and digital participation, with implications for integrated community mental health policy in China.

## Introduction

China is aging at a pace few societies have experienced. By the middle of this century, older adults will account for a substantial share of the national population, and late-life mental health has become a central concern for health systems, families, and social policy (1). Depressive symptoms are common in this period of life. They reduce quality of life, weaken treatment adherence, and increase care dependence (2, 3). In the Chinese context, rapid social change, uneven community resources, and persistent urban–rural disparities may intensify emotional vulnerability among older adults who also carry historical burdens from earlier decades of material scarcity and family disruption. Analyses based on the China Longitudinal Aging Social Survey have already shown that depressive symptoms among older adults are socially patterned and frequently co-occur with chronic disease (4), underscoring the need to locate mental health risk within broader aging trajectories rather than treating it as an isolated clinical event.

A growing body of research has linked adverse childhood experiences to poorer mental health in adulthood and later life. Classic and contemporary evidence indicates that early adversity, including abuse, household dysfunction, hunger, constrained medical access, and parental loss, predicts depression and other health problems across the life course (5–7). In China, CHARLS-based studies have likewise documented a “long arm” of childhood adversity for middle- and later-life depression, including dose–response associations and elevated odds of unfavorable depressive symptom trajectories (8–10). Recent mediation work further suggests that contemporary health conditions, such as sleep disruption and chronic disease burden, transmit part of the ACE-depression association in older Chinese adults (11). Yet many analyses still treat childhood adversity and late-life depression as distant correlates rather than connected phenomena with identifiable present-day channels. This gap carries policy relevance: although childhood adversity cannot be reversed, current health status, social adaptation, and digital engagement remain amenable to service design.

These associations should also be read against twentieth-century Chinese history. Many respondents in CLASS 2023 spent childhood during or after the 1959–1961 famine and, for older cohorts, during the Cultural Revolution, periods that prior work has linked to later health, educational disruption, and mental health risk (12–15). Period-level shocks may therefore raise the average later-life risk in this generation and may confound or moderate estimated adversity–depression associations. Two features of the present design nevertheless keep the individual-level gradient interpretable. First, hunger, medical deprivation, and parental loss still vary among respondents who share the same historical period; that within-cohort variation is what the adversity score uses. Second, in a single 2023 wave, birth year and chronological age are collinear, so a famine or Cultural Revolution cohort interaction would largely recast the age adjustment already included in the models rather than identify a distinct historical treatment. The present study therefore locates these events as Introduction context. It cannot separate individual-level childhood hardship from cohort-level historical exposure, and it does not treat a formal historical-moderation test as a prerequisite for the parallel-mediation estimates.

Life-course and cumulative disadvantage perspectives help explain why early hardship may still matter decades later. Cumulative dis/advantage theory emphasizes that inequalities tend to widen over time through interconnected risks and resources, while remaining open to modification by later-life conditions and agency (16). In that framing, childhood adversity is not merely a remote psychological scar; it may shape health capital, social competence, and access to contemporary coping resources that, in turn, structure depressive risk in old age. Three present-day domains are especially relevant in aging China. Self-rated health captures perceived physical decline and functional confidence and is consistently associated with depressive symptoms in older populations; longitudinal Chinese evidence shows reciprocal links between self-rated health and depression over the later life course (17). Internet media use has become increasingly important as communication, information, and service access move online. Limited engagement may narrow practical coping resources and social connection, a pattern reinforced by recent Chinese studies linking multidimensional internet or digital technology use to lower depressive burden, partly through social participation (18–22). Social adaptation reflects perceived difficulty fitting into changing social environments. Unlike broad network counts, adaptation strain speaks directly to everyday emotional stress in aging communities, and related work on social support, cohesion, isolation, and loneliness shows that social fit and connection are central to late-life mental health (23–27). Social adaptation is a multidimensional construct that can include social-role adjustment, reconstruction of relationships, and willingness to participate (28, 29). The four CLASS items used here capture perceived difficulty adapting to rapid social change, new ideas, new policies, and age-unfavorable social change, rather than the full construct. We therefore interpret this mediator as adaptation strain, not as a complete measure of social functioning.

Prior Chinese studies have examined depression alongside health, social relations, or digital behavior, but few have integrated childhood adversity with multiple parallel pathways in one nationally representative older-adult sample. Research based on CHARLS and regional cohorts has shown that depressive symptoms are widespread and socially patterned, and that residential and family context can shape how emotional distress relates to other aging outcomes. Empty-nest status and social alienation are particularly salient in Chinese aging research: meta-analytic and community studies report high depression prevalence among empty nesters and highlight alienation, loneliness, and support deficits as important correlates (30, 31). Zhao et al. (32), for example, demonstrated that depressive symptoms were associated with cognitive performance among Chinese older adults and that residential region partially mediated this relationship, with stronger patterns among empty-nesters. That line of work underscores how aging outcomes are embedded in place, family structure, and social context. Stereotype and social-perception processes may further color how older adults interpret aging and social change (33, 34), making adaptation strain a theoretically meaningful pathway alongside health and digital participation. The present study extends this perspective by shifting attention from cognition alone to depressive symptoms and from residential mediation alone to a broader set of present-day pathways linked to health, adaptation, and digital participation, which are pathways that parallel mediation can estimate simultaneously.

The present study addresses this gap using CLASS 2023 data. We examine how childhood adversity relates to depressive symptoms in later life, how much of that relationship is statistically accounted for by self-rated health, internet media use, and social adaptation when these concurrent correlates are estimated simultaneously, and whether the adversity–depression association differs across social contexts that are central to aging policy—community activity participation, urban–rural residence, and empty-nest family structure. By situating early-life disadvantage within modifiable contemporary conditions, the study contributes China-specific evidence on where community mental health interventions may interrupt adversity-linked depressive burden.

We organized the analyses around three hypotheses implied by the life-course framework and Figure 1. *H1*: childhood adversity is positively associated with later-life depressive symptoms. *H2*: poorer self-rated health, less frequent internet media use, and poorer social adaptation jointly account for a substantial share of that association in a parallel decomposition, and each specific indirect association is expected to be nonzero, with social adaptation and self-rated health expected to be the larger concurrent correlates. *H3*: the adversity–depression association is stronger among urban older adults, those with lower community participation, and non-empty-nest respondents. These hypotheses specify expected directions of association; they do not imply that the cross-sectional design can identify causal effects.

**Figure 1:**
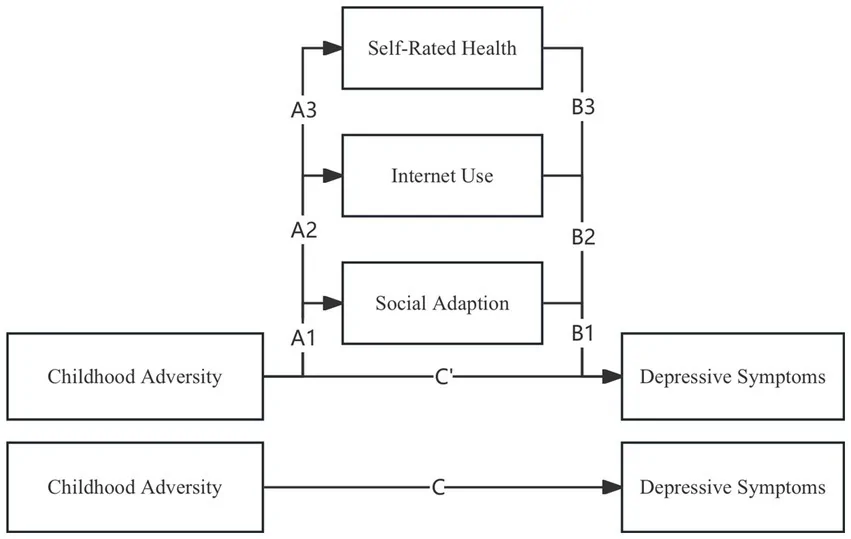
Variable relationships. Path A1 to A3 represents the Association between Childhood Adversity and the Potential 3 Mediators. Path B1 to B3 stands for the correlation between the potential 3 Mediators and Depressive Symptoms. Path C is the association between Childhood Adversity and Depressive Symptoms. Path C′ represents the Direct Associations of Childhood Adversity on Depressive Symptoms.

## Methods

### Data source and sample selection

This study used individual-level data from the 2023 wave of the China Longitudinal Aging Social Survey (CLASS), a nationally representative survey of older adults designed to capture health, family, social participation, and digital behavior. CLASS applies probability sampling across 28 provincial-level regions, with post-stratification weights for urban–rural residence, sex, and age. The public 2023 individual file contains 11,670 respondents. An official fieldwork response rate is not reported in the released file; published CLASS documentation describes a nationally representative design covering 28 provincial-level regions, with follow-up waves generally maintaining a sample on the order of 12,000 respondents.

The descriptive sample in Table 1 uses the released file (*N* = 11,670). Inferential models used complete cases. After listwise deletion of missing covariates, 10,480 observations entered the continuous total-effect model in Table 2 (10,476 for the binary sensitivity model). Parallel mediation further required complete data on all three mediators, yielding *N* = 10,093 in Tables 3–5. Complete-case analysis can introduce selection if missingness is related to health or social resources; we therefore report sample sizes for each model and interpret estimates as applying to respondents with complete information on the variables in that model. Analyses incorporated survey weights normalized to the sample mean.

**Table 1:** Descriptive statistics of variables (*N* = 11,670).

Variable	Overall	Younger-old (60–74)	Older-old (75+)
Sample size, *n*	11,670	8,813	2,835
Depression prevalence, %	32.9	31.4	41.7
CES-D total score, mean (SD)	6.25 (3.44)	6.09 (3.47)	7.22 (3.12)
Childhood adversity total, mean (SD)	1.56 (0.92)	1.53 (0.93)	1.73 (0.80)
Self-rated health, mean (SD)	2.37 (0.79)	2.32 (0.79)	2.60 (0.77)
Internet media use, mean (SD)	2.85 (1.69)	3.02 (1.67)	1.85 (1.40)
Social adaptation, mean (SD)	12.06 (3.38)	12.05 (3.40)	12.13 (3.25)
Age (years), mean (SD)	69.52 (4.93)	68.08 (3.59)	77.82 (2.97)
BMI, mean (SD)	23.20 (2.55)	23.29 (2.53)	22.73 (2.59)
Co-residents, mean (SD)	2.53 (1.23)	2.52 (1.21)	2.61 (1.31)
Female, %	40.7	40.9	40.0
Married/cohabiting, %	87.0	89.4	73.4
Rural residence, %	44.3	43.0	51.9
Empty-nest, %	76.6	78.2	67.1
Education: low, %	17.0	14.4	31.9
Education: primary, %	35.7	34.4	43.3
Education: middle+, %	47.3	51.2	24.7
Income: better, %	11.9	12.1	11.2
Income: about same, %	80.5	80.7	79.6
Income: worse, %	7.6	7.3	9.2

Higher scores on self-rated health and social adaptation indicate poorer health and poorer adaptation. Depression prevalence is reported descriptively using the conventional CES-D ≥ 5 cutoff and was not used in inferential models.

**Table 2:** Main-effect regression models on childhood adversity with depressive symptoms.

Variable	Continuous CES-D outcome		Binary CES-D outcome		
	b	SE	b	SE	OR
Childhood adversity total	0.439^***^	0.043	0.268^***^	0.028	1.308
Age	0.061^***^	0.007	0.049^***^	0.005	1.051
Gender	0.137^*^	0.070	0.070	0.048	1.072
Educational attainment	−0.419^***^	0.051	−0.293^***^	0.037	0.746
Marital status	−0.710^***^	0.113	−0.465^***^	0.094	0.628
Household economic status	0.438^***^	0.079	0.264^***^	0.055	1.302
Rural residence	0.582^***^	0.075	0.327^***^	0.052	1.387
Empty-nest	−0.584^**^	0.178	−0.374^**^	0.134	0.688
Number of co-residents	−0.036	0.060	−0.043	0.046	0.958
BMI	−0.086^***^	0.014	−0.041^***^	0.009	0.960
Constant	4.275^***^	0.727	−1.253^*^	0.526	
*N*	10,476		10,476		
*R* ^2^	0.092		0.066		
Adj. *R*^2^	0.091		0.066		

Due to missing values in covariates, the regression sample size is 10,476 for the continuous total-effect model and 10,476 for the binary model. These models do not include the three mediators. The 0.439 coefficient is therefore not directly comparable to the 0.348 total association in Tables 3–5, which is estimated on the complete-mediator sample (*N* = 10,093). *p < 0.05, **p < 0.01, ***p < 0.001. OR, odds ratio.

**Table 3:** Associations of childhood adversity on depressive symptoms mediated by social adaptation among older adults in China (*N* = 10,093).

Variable	Social adaptation		Depressive symptoms		Mediated effect		
	b	SE	b	SE	b	SE	%
Social adaptation			0.229	0.010			
Childhood adversity total	0.641	0.043	0.348	0.044	0.147^**^	0.012	42.2
Childhood adversity direct			0.026	0.039			

Models adjusted for sex, age, education, marital status, relative economic status, urban–rural residence, and empty-nest status. Bootstrap confidence intervals based on 1,000 replications. **p < 0.01.

**Table 4:** Associations of childhood adversity on depressive symptoms mediated by self-rated health among older adults in China (*N* = 10,093).

Variable	Self-rated health		Depressive symptoms		Mediated effect		
	b	SE	b	SE	b	SE	%
Self-rated health			0.966	0.045			
Childhood adversity total	0.129	0.009	0.348	0.044	0.125^**^	0.010	35.7
Childhood adversity direct			0.026	0.039			

Models adjusted for sex, age, education, marital status, relative economic status, urban–rural residence, and empty-nest status. Bootstrap confidence intervals based on 1,000 replications. **p < 0.01.

**Table 5:** Associations of childhood adversity on depressive symptoms mediated by internet media use among older adults in China (*N* = 10,093).

Variable	Internet media use		Depressive symptoms		Mediated effect		
	b	SE	b	SE	b	SE	%
Internet media use			−0.386	0.024			
Childhood adversity total	−0.132	0.018	0.348	0.044	0.051^**^	0.008	14.7
Childhood adversity direct			0.026	0.039			

Models adjusted for sex, age, education, marital status, relative economic status, urban–rural residence, and empty-nest status. Bootstrap confidence intervals based on 1,000 replications. **p < 0.01.

### Measures

Childhood adversity was constructed as a cumulative exposure index ranging from 0 to 4, summing three indicators: experience of childhood hunger (yes = 1), inadequate childhood medical care (yes = 1), and parental loss by age 10 (both parents alive = 0, one parent deceased = 1, both parents deceased = 2). The parental-loss component is a count of deceased parents by age 10, not an arbitrary double-weighting of a binary indicator relative to hunger or medical deprivation. Higher scores indicated greater early-life disadvantage. The three indicators are heterogeneous in content, so the composite is treated as an index of cumulative early-life exposure rather than a unidimensional latent scale (Cronbach α = 0.31, reported only as a heterogeneity diagnostic). CLASS 2023 does not record the age at parental loss, the duration of hunger, or childhood socioeconomic status separate from these items; those omissions are discussed as limitations. As a supplementary check, we also estimated indicator-specific associations and a 0–3 composite in which parental loss was recoded as any versus none.

Depressive symptoms were measured with a nine-item short form of the Center for Epidemiologic Studies Depression Scale (CES-D). Respondents reported symptom frequency on a three-point scale. Positive-affect items were reverse-coded so that higher total scores indicated greater symptom burden. Internal consistency for the nine items was acceptable (Cronbach α = 0.73). The primary outcome was continuous CES-D total score (range 0 to 18) used to report depression prevalence. The cutoff value is 5 related to previous research results and the Kohout’s formula (36, 47, 49). The short-form CES-D is a symptom screen and does not constitute a clinical diagnosis of major depressive disorder.

Three parallel mediators were examined. Self-rated health was a single item on current health status (1 = very healthy to 5 = very unhealthy), with higher scores indicating poorer health. Internet media use was a single item on frequency of internet use, including mobile internet, during the past 3 months (1 = never to 5 = always). Because both are single items, internal consistency cannot be estimated, and measurement error may attenuate associations with childhood adversity (path a) and/or with depressive symptoms (path b), which can bias indirect associations toward or away from zero depending on which path is more affected. Social adaptation was the sum of four CLASS items (each 1 = completely inconsistent to 5 = completely consistent): “Social changes are happening so fast that I find it difficult to adapt to them”; “More and more opinions nowadays are difficult for me to accept”; “More and more new social policies today are difficult for me to accept”; and “Current social changes are increasingly unfavorable to older people.” Higher summed scores indicated poorer adaptation (Cronbach α = 0.79; range 4–20). These items, drawn from CLASS’s social-adaptation module based on Chen (29), index perceived difficulty adapting to social change rather than social-role occupancy, relationship reconstruction, or participation willingness. Content validity is therefore partial relative to the broader construct.

As for covariates, models adjusted for sex, age, education, marital status, relative household economic status, urban–rural residence, and empty-nest status. Sex was coded as male or female. Age was measured in years and also grouped into 60–69, 70–79, and 80 or older for descriptive stratification. Education was grouped into low (illiterate or limited literacy), primary school, and middle school or above. Marital status was dichotomized into married or cohabiting versus not married. Relative economic status captured the household’s perceived economic position relative to local peers and was grouped into better, about the same, and worse. Urban–rural residence followed the survey classification. Empty-nest status identified respondents living alone or with a spouse only, without co-resident children.

### Statistical analysis

All estimates are associations. Mediation analysis conceptually assumes a temporal order among the independent variable, mediators, and outcome, but CLASS 2023 is a single wave. Childhood adversity is retrospectively reported and is temporally prior by construction, whereas self-rated health, internet media use, social adaptation, and depressive symptoms were measured concurrently and may be bidirectionally related or share common method variance. Depressive symptoms may lead respondents to evaluate health and social adaptation more negatively and may reduce internet use. We therefore interpret parallel mediation as a decomposition of the observed association, not as evidence that the mediators are causal channels. Weighted ordinary least squares regression was used for the continuous CES-D outcome. Weighted logistic regression was used for binary depression outcomes in sensitivity analyses. Parallel mediation followed a path decomposition framework in which childhood adversity (X) was related to each mediator (M) and to depressive symptoms (Y) while all mediators and covariates were included simultaneously. This approach extends the logic of Baron and Kenny's (35) mediation strategy to a multiple-mediator setting in which specific indirect associations can be estimated for each pathway while the others are held constant. The parallel specification assumes that the mediators do not cause one another; modest correlations among mediators (*r* = −0.25 to 0.14) make that working assumption more plausible but do not test serial or reciprocal relations. We used bootstrap resampling rather than Sobel tests alone because indirect effects are products of coefficients and are not normally distributed in finite samples. The specific models are as follows: The specific models are given in Equations 1–3.


Y=c⋅X+controls+e (1)

$$M_{i}=a_{j}\cdot X+\text{controls}+e_{j}$$ (2)

$$\begin{array}{c} Y=c^{\prime }\cdot X+b_{1}\cdot M_{1}+b_{2}\cdot M_{2}+b_{3}\cdot M_{3}+\text{controls}+e \end{array}$$ (3)

Where $M_{1}$ , $M_{2}$ , and $M_{3}$ denoted self-rated health, internet media use, and social adaptation. Specific indirect effects were calculated as $a_{j}$ × $b_{j}$ . Bootstrap resampling (1,000 replications) generated percentile confidence intervals for indirect effects.

False Discovery Rate (FDR) method was applied across mediator-specific tests (47–49). Subgroup analyses stratified the adversity-depression association by community activity participation, urban–rural residence, and empty-nest status, reporting odds ratios for binary depression and regression coefficients for continuous CES-D. Those comparisons require the CES-D-9 to measure the same construct across groups. We therefore tested two-factor measurement invariance of the nine ordinal items [negative affect: six items; positive affect: three reverse-coded items; (36)] across sex, urban–rural residence, and age (60–74 versus 75 years or older). We estimated configural, metric, and scalar models using the weighted least squares mean- and variance-adjusted estimator (WLSMV) and theta parameterization, identified following Wu and Estabrook (37). Metric invariance constrained thresholds and loadings; scalar invariance further constrained intercepts. Invariance was judged by change in comparative fit and RMSEA, using |ΔCFI| < 0.010 and |ΔRMSEA| < 0.015 (38, 39). Full estimates are reported in Supplementary Tables S1 and S2. Variance inflation factors in the parallel outcome model were low for childhood adversity and the three mediators (VIF = 1.06–1.46), indicating no serious collinearity among the hypothesized concurrent correlates. Empty-nest status and number of co-residents had higher VIFs (5.32 and 4.97), as expected given their conceptual overlap; both were retained because they capture related but distinct household features. A Harman single-factor check on the core self-report items explained 21.6% of variance, below the conventional 40% rule of thumb, but common-method bias cannot be ruled out in a single-wave questionnaire. Retrospective childhood reports may be influenced by current mood. Analyses were conducted in Python 3 with statsmodels. Measurement-invariance models were estimated in R using lavaan.

## Results

### Descriptive statistics

Table 1 presents descriptive characteristics of the analytic sample. Respondents had a mean age of 69.5 years (SD = 4.9). The mean CES-D score was 6.25 (SD = 3.44), and 32.9% of respondents scored at or above the conventional short-form cutoff of 5. The mean childhood adversity score was 1.56 (SD = 0.9), indicating that hunger, medical deprivation, and parental loss were common historical experiences in this cohort. Mean social adaptation score was 12.06 (SD = 3.38), with higher values reflecting poorer adaptation.

Childhood adversity was modestly correlated with poorer self-rated health (*r* = 0.15), lower internet media use (*r* = −0.17), and poorer social adaptation (*r* = 0.20). Correlations among the three mediators were small to moderate. Self-rated health correlated with internet media use at *r* = −0.25 and with social adaptation at *r* = 0.14. Internet media use and social adaptation correlated at *r* = −0.14. This pattern suggests related but distinguishable pathways rather than redundant measures of the same construct (Figure 2).

**Figure 2:**
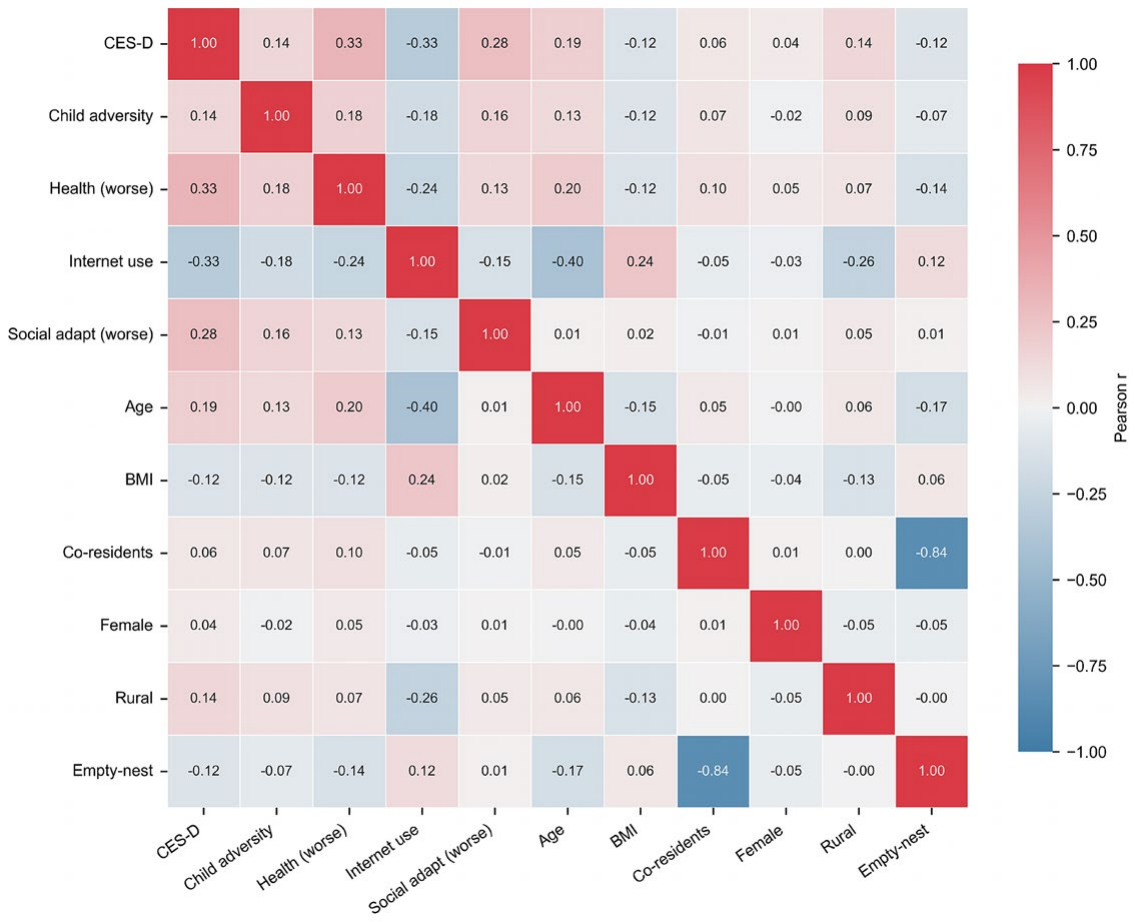
Correlation heatmap of core variables.

### Associations of childhood adversity with depressive symptoms

Childhood adversity was positively associated with depressive symptoms in the total-effect model. Each one-unit increase in the adversity score was associated with a 0.439-point increase in CES-D score (*p* < 0.001; *N* = 10,476). Relative to the sample standard deviation of 3.44, this is a small individual-level increment (about 0.13 SD per unit). In logistic models using the binary outcome, the corresponding association was also positive and statistically significant (OR = 1.308, 95% CI: 1.237–1.382, *p* < 0.001), confirming that the adversity-depression link was robust to different specifications of the outcome. Patterns for covariates were generally consistent with prior research: older age, female sex, lower education, worse perceived economic status, and rural residence were associated with greater depressive symptom burden, though some coefficients differed between continuous and binary models.

Collectively, these total-effect results establish a robust adversity-depression association that persists after adjusting for a comprehensive set of sociodemographic covariates. The consistency across continuous and binary outcome specifications strengthens confidence in the observed relationship and provides a solid foundation for the subsequent mediation decomposition.

When the three indicators were entered simultaneously in the same covariate-adjusted sample, each remained positively associated with CES-D: medical deprivation (b = 0.642, 95% CI 0.454–0.830), hunger (b = 0.420, 95% CI 0.267–0.572), and parental loss coded 0–2 (b = 0.232, 95% CI 0.096–0.367). Recoding parental loss as any versus none and summing a 0–3 index yielded a total association of 0.468, close to the original 0–4 composite (0.439). These checks suggest that the main association is not an artifact of counting two deceased parents as two points, although the indicators are not interchangeable in magnitude.

### Parallel mediation analysis

After including the three mediators simultaneously in the complete-mediator sample (*N* = 10,093), the total association between childhood adversity and CES-D was 0.348, smaller than the 0.439 estimate in Table 2 because of the additional complete-case restriction. The remaining direct association was 0.026 (*p* = 0.464). The three specific indirect associations summed to 0.323, or about 92% of the 0.348 total in this specification. This percentage is a model-dependent descriptive ratio, not a causal share of “channels” through which adversity operates, and it can move with sample definition, covariate set, and measure reliability.

The indirect association through social adaptation was 0.147 (bootstrap SE = 0.012). Childhood adversity predicted poorer social adaptation (a = 0.641), and poorer adaptation predicted higher CES-D scores (b = 0.229). This was the largest specific indirect association in the parallel model.

The indirect association through self-rated health was 0.125. Adversity was associated with poorer self-rated health (a = 0.129), and poorer health predicted higher CES-D scores (b = 0.966). In proportional terms, health and social adaptation accounted for similar shares of the decomposed association. A bootstrap contrast of the two specific indirect associations was 0.022 (95% CI − 0.007 to 0.055) and did not exclude zero, so the data do not support a claim that the social-adaptation path is statistically larger than the health path. Both were larger than the internet-media indirect association (social minus internet = 0.096, 95% CI 0.071–0.120; health minus internet = 0.073, 95% CI 0.048–0.098).

The indirect association through internet media use was 0.051 (bootstrap SE = 0.008). Adversity predicted lower internet media use (a = −0.132), and lower use predicted higher CES-D scores (b = −0.386). Although smaller than the health and adaptation associations, this digital association remained statistically nonzero in the parallel specification.

### Subgroup analyses

Scalar invariance of the CES-D-9 was supported, so subgroup differences in CES-D associations can be compared as differences in the same construct rather than as artifacts of group-specific item functioning. We estimated two-factor WLSMV models by sex (*N* = 10,502), urban–rural residence (*N* = 10,502), and age 60–74 versus 75 years or older (*N* = 10,480). From the configural to the scalar model, scaled ΔCFI was −0.0009 for sex, 0.0012 for residence, and 0.0008 for age; scaled ΔRMSEA was −0.0061, −0.0038, and −0.0039, respectively (Supplementary Table S2). All absolute changes are far below 0.010 for ΔCFI and 0.015 for ΔRMSEA. Nested chi-square tests were nonsignificant for sex (*p* = 0.173) and significant for residence and age; with *N* ≈ 10,500, chi-square detects trivially small discrepancies and is not the decision rule. These results justify the CES-D subgroup comparisons below. The association between childhood adversity and depressive symptoms varied considerably across social contexts. Geographically, the association was pronounced among urban older adults (*β* = 0.507, *p* < 0.001) but was absent in rural areas (*β* = 0.105, *p* = 0.152). In the main-effect sample, mean adversity was higher in rural than urban respondents (1.63 vs. 1.44) and the rural standard deviation was somewhat smaller (0.81 vs. 0.92). Restricted rural variance may contribute to the null rural estimate but is modest and cannot by itself establish the mechanism. Socioeconomic stratification also mattered: the effect was largest among respondents with middle school education or above (*β* = 1.046, *p* < 0.001), a relatively small subgroup, and grew monotonically with worsening household economic status, from *β* = 0.465 among better-off respondents to *β* = 0.692 among worse-off respondents.

Behavioral and social participation further modified the association. Among older adults who did not vote in community elections, the adversity-depression link was particularly strong (*β* = 0.805, *p* < 0.001), whereas it was negligible among those who were not eligible to vote (*β* = 0.023, *p* = 0.807) and smaller, but still positive, among those who did vote (*β* = 0.147, *p* = 0.049). This voted-group coefficient is smaller but still positive and should not be interpreted as a protective or buffering effect of community participation. Digital engagement revealed a similar gradient: the association was roughly twice as large among high-engagement respondents (*β* = 0.394) as among low-engagement respondents (*β* = 0.182).

Demographic and family structure patterns also emerged. The association was significant among adults aged 60–69 and 70–79 but was not significant in the oldest-old group (aged 80+, *β* = −0.016, *p* = 0.901). The 80 + stratum is comparatively small (*n* = 1,070), so the null estimate may reflect limited precision, selective survival of more resilient respondents, or both; the present design cannot distinguish these explanations. Women exhibited a stronger association (*β* = 0.450) than men (*β* = 0.293). Regarding family composition, the association was larger among non-empty-nest older adults (*β* = 0.579) than among empty-nest counterparts (*β* = 0.303). This contrast is the opposite of a simple empty-nest vulnerability story and should be treated as descriptive heterogeneity, not as evidence that co-residence is harmful or protective.

## Discussion

Using nationally representative data, this study found a clear association between childhood adversity and depressive symptoms in Chinese older adults. The central finding is that this association was statistically accounted for largely by present-day self-rated health, social adaptation, and internet media use. When these three measures were entered in parallel, the remaining direct association of adversity became negligible. Late-life depressive symptoms in this cohort were therefore less a standalone emotional residual of distant hardship in the statistical decomposition, and more a manifestation of how early disadvantage co-occurs with current health and social functioning. This pattern is consistent with cumulative disadvantage reasoning: early exposures matter because they are linked to later distributions of health capital, social fit, and digital participation, not only because they leave an immutable direct scar (8, 16). The 92% ratio should be read cautiously: it is large in this specification, is sensitive to sample and measurement, and cannot establish that the mediators are the mechanisms through which adversity produces depression.

What the present findings add is a clearer map of how that historical exposure appears to matter in a contemporary older-adult CLASS sample. The adversity–depression link was not primarily a residual direct association once the three concurrent measures were included. After these mediators were entered simultaneously, the nonsignificant direct path should not be read as evidence that childhood adversity is unimportant; rather, it indicates that adversity-related mental health burden in later life is statistically concentrated in present-day conditions that may be more amenable to services. A high mediated proportion in cross-sectional data can also reflect common method bias or conceptual overlap: some social-adaptation items may share evaluative or affective content with depressive symptoms, and self-rated health may overlap with somatic complaints on the CES-D. Those possibilities caution against treating the 92% figure as a substantive claim that almost all of the effect “passes through” the three mediators.

The social adaptation association deserves particular attention. In the parallel model, it showed the largest specific indirect association. Older adults with greater childhood adversity reported more difficulty adapting to social change, and that adaptation strain was strongly associated with depressive symptoms. This result extends prior work on social integration and late-life mental health by highlighting adaptation difficulty as a distinct emotional mechanism. Cramm et al. (40) and Yu et al. (41) have emphasized neighborhood cohesion and social context as resources for older adult well-being, while broader evidence links social isolation and loneliness to adverse health outcomes (24, 26). In China, social isolation has been associated with depression risk among older adults in recent population surveys (27), and studies of empty-nest and rural older adults show that social alienation and weak support are tightly intertwined with depressive symptoms (30, 31). Adaptation strain captures a related but distinct dimension: not simply whether social ties exist, but whether older adults feel able to fit into changing social environments. In rapidly aging Chinese communities, where neighborhood restructuring, intergenerational co-residence, and shifting social roles are common, this distinction may be especially important. Bai (23) likewise shows that social adaptation helps organize the link between support and depressive symptoms among Chinese older adults, reinforcing the value of treating adaptation as a central psychosocial pathway.

Life-course theory offers two complementary accounts of why early adversity might be linked to later adaptation strain. A sensitive-period account emphasizes disruption of early attachment and trust when hunger, medical deprivation, or parental loss occurs in childhood (42). An accumulation account emphasizes progressive depletion of coping resources and social competence across adulthood (16). Social-cognitive perspectives similarly suggest that early hardship can shape later efficacy beliefs and expected social threat (43). Continuity theory further implies that difficulty reconciling past identities with rapid social change can itself be a source of late-life strain (28). The present 4-item measure captures perceived mismatch with contemporary social change rather than attachment or efficacy directly, so these accounts remain interpretive. Because the mediators were modeled in parallel, we also cannot test whether poorer social adaptation and lower internet use form a serial or reciprocal loop; that possibility is a direction for longitudinal research.

The health association remained strong and consistent with life-course accumulation accounts. Childhood adversity predicted poorer self-rated health, which in turn predicted higher CES-D scores. Grossman’s (44) health capital framework and subsequent gerontological research both suggest that early disadvantage can erode health over decades, and perceived health remains one of the strongest correlates of late-life depression (2, 17). Recent Chinese mediation evidence similarly identifies contemporary physical health burdens as transmitters of ACE-related depressive risk (8, 11). The present results suggest that part of that burden among adversity-exposed older adults may be carried through current perceived health. This supports integrated models of aging care in which mental health screening is embedded in chronic disease management and functional assessment (1). Depression prevention in older adults should not be separated from routine health follow-up in primary and community settings.

That health path should be interpreted as a global perceived-health correlate, not as an identified biomedical sequence. Childhood adversity may affect later self-rated health through accumulated chronic disease, functional limitation, pain, and sleep problems; self-rated health is a subjective composite of these more specific burdens. The CLASS single item cannot decompose those internal physiological and functional routes, so the present model can treat self-rated health only as an overall health-perception mediator. Li et al. (11) show that chronic disease and sleep disorders mediate part of the ACE–depression association among older Chinese adults, a finding consistent with the self-rated-health path estimated here. Future research can test a sequential specification—childhood adversity → chronic disease, functional limitation, or pain → self-rated health → depressive symptoms—without implying that the current parallel model already isolates those nested mechanisms.

The internet media association was smaller but stable. Adversity was associated with less frequent internet media use, and lower use was associated with greater depressive symptoms. In contemporary China, where information, communication, and many services are digitally mediated, limited media engagement may reduce practical support and widen everyday exclusion. Van Deursen and van Dijk (45) argued that the digital divide has shifted from access alone to differences in usage and competence; among older adults, limited media participation may therefore matter more than whether a device is available at all. Chen and Schulz (46) reviewed evidence that communication technology can reduce social isolation in later life, although effects depend on meaningful use rather than superficial contact. Recent Chinese evidence strengthens this interpretation: multidimensional internet use is associated with lower depression partly through social participation (18, 22), internet use and digital engagement relate to depressive symptoms in CHARLS samples (19, 20), and persistent access and usage divides remain linked to poorer healthy aging outcomes, including depressive symptoms and social isolation (21). The present findings fit that view. The digital pathway was smaller than health and adaptation, yet it remained significant after parallel adjustment. Digital inclusion policies should therefore emphasize sustained media participation and practical online competence, not only basic connectivity. The comparatively small internet-media association may also reflect measurement: a single frequency item over 3 months cannot distinguish information seeking, social communication, and service use, so attenuation from coarse measurement is plausible. Future work should use multidimensional digital-engagement measures.

Subgroup findings add an important layer of interpretation and echo recent Chinese aging research showing that the expression of emotional risk depends on social context. Two-factor WLSMV tests supported scalar invariance of the CES-D-9 by sex, urban–rural residence, and age: configural-to-scalar |ΔCFI| was at most 0.0012 and |ΔRMSEA| at most 0.0061, both far below 0.010 and 0.015 (Supplementary Table S2). Chi-square difference tests were significant for residence and age because of the large sample; they do not overturn the approximate-fit conclusion. Table 6 subgroup comparisons of CES-D associations are therefore appropriate on measurement grounds. The adversity–depression association was strongest among urban, low-activity, and non-empty-nest older adults. Among respondents who voted in community elections, the association was weaker (*β* = 0.147) than among those who did not vote (*β* = 0.805), but it remained positive and should not be interpreted as a reversal or as a buffering effect of community participation. Reverse causation is equally plausible: older adults with fewer depressive symptoms may be more able to participate, and participation may also mark better health and socioeconomic resources that are themselves related to childhood adversity. We therefore treat the participation contrast as exploratory heterogeneity, not as evidence that community activity interrupts a causal chain.

**Table 6:** Subgroup associations between childhood adversity and depressive symptoms.

Subgroup	Stratum	*n*	*β*	*p* (FDR)
Sex	Female	4,892	0.450	<0.001
Sex	Male	5,205	0.293	<0.001
Age	60–69	4,092	0.387	<0.001
Age	70–79	4,935	0.331	<0.001
Age	80+	1,070	−0.016	0.901
Residence	Urban	5,731	0.507	<0.001
Residence	Rural	4,366	0.105	0.152
Education	Low	2,063	0.238	0.029
Education	Primary	6,601	0.135	0.020
Education	Middle+	1,433	1.046	<0.001
Income	Better	1,137	0.465	0.002
Income	About same	8,171	0.321	<0.001
Income	Worse	789	0.692	<0.001
Empty-nest	Empty-nest	7,534	0.303	<0.001
Empty-nest	Non-empty-nest	2,563	0.579	<0.001
Civic participation	Voted	3,675	0.147	0.049
Civic participation	Did not vote	3,439	0.805	<0.001
Civic participation	Not eligible	2,983	0.023	0.807
Digital engagement	High	5,019	0.394	<0.001
Digital engagement	Low	5,078	0.182	0.007

ORs based on logistic models with depression defined as CES-D ≥ 5. All models adjusted for covariates. p-values for subgroup analyses were adjusted for multiple comparisons using the False Discovery Rate (FDR) method; significance was determined at the FDR-corrected threshold.

The urban–rural contrast also merits careful reading. Adversity was significantly associated with depression in urban areas but not in rural areas in the stratified models. Rural respondents had a higher mean adversity score and a somewhat smaller standard deviation, which can reduce estimated associations, but the variance difference is not large. Other interpretations remain open: rural survivors in a national survey may be a selectively resilient group; adversity-related distress in rural settings may be expressed through unmeasured pathways such as physical labor continuity, informal family support, or different symptom norms; and urban social comparison, service expectations, and faster environmental change may make early disadvantage more emotionally salient. Empty-nest research further cautions against assuming a single rural or urban template: depression among empty nesters is common across settings, but mechanisms such as alienation and support deficits may operate differently by place (30, 31). Any of these explanations would imply that uniform national programs are unlikely to be equally effective across residence contexts.

The family-structure contrast indicates that co-residence does not automatically confer protection against adversity-linked depression. Adversity associations were larger among non-empty-nest older adults than among empty-nest older adults, although both were statistically significant. Possible explanations include intergenerational tension, household care burden, or unmet emotional expectations within multigenerational living arrangements, but the present data do not measure relationship quality, caregiving load, or expected support, so these accounts are speculative. Aging policy in China has often emphasized family support as a foundation of care for older adults, and that emphasis remains important. Yet household composition alone does not determine emotional risk. Meta-analytic work on empty-nest depression and studies of social alienation among rural empty-nest older adults both imply that relational quality and perceived social fit matter at least as much as living arrangement labels (30–32). Services for co-residing older adults may therefore need to address family interaction strain and role adjustment, not only material support.

These findings should be read alongside several limitations. First, the data are cross-sectional, so temporal ordering among mediators and depressive symptoms cannot be established and mediation results represent association decomposition rather than causal mechanisms. Subgroup contrasts may also reflect residual confounding and compositional differences across strata. Second, self-rated health, social adaptation, internet use, and depression may be reciprocally related, as suggested by longitudinal work on self-rated health and depression (17). Third, childhood adversity is retrospectively self-reported and may be influenced by current emotional state. Fourth, CLASS primarily represents community-dwelling older adults and does not fully capture institutionalized or severely disabled populations. Fifth, CLASS 2023 does not include an independent childhood socioeconomic measure such as parental education or childhood household income. Hunger and inadequate medical care partly capture material deprivation, so omitted childhood SES would most likely bias the adversity coefficient upward if early poverty jointly produced both the measured items and later depression. Adult education, marital status, and economic status absorb part of this pathway, but they do not fully block it. Residual confounding of this kind would threaten a causal interpretation of the total association more than it would erase the finding that concurrent health, adaptation, and digital measures statistically account for most of the observed link. Sixth, genetic factors and personality traits such as neuroticism were not measured and could inflate both recalled adversity and CES-D, again biasing the total association upward. The near-zero remaining direct association after the three concurrent measures are entered suggests that any such confounding would also have to operate largely through present-day conditions rather than as a large residual direct path. Seventh, measurement invariance of the CES-D-9 was tested rather than assumed. Two-factor WLSMV models supported scalar invariance across sex, urban–rural residence, and age: configural-to-scalar |ΔCFI| and |ΔRMSEA| were far below 0.010 and 0.015 (Supplementary Table S2). Significant chi-square tests for residence and age reflect sample size, not a material violation. Subgroup comparisons of CES-D in Table 6 are therefore appropriate. Self-rated health is a single item, so loading invariance does not apply. Residual confounding and compositional differences across strata remain possible even when measurement is invariant. Eighth, severity and duration of childhood exposures (for example, age at parental loss or length of hunger) are not available in CLASS 2023. Ninth, the short-form CES-D is a screening score, not a clinical diagnosis; a 0.44-point increment per adversity unit is small relative to the 3.44-point standard deviation, and no established minimal clinically important difference exists for this 9-item 0–18 CLASS version, so population-level public health relevance should not be equated with individual clinical importance. Tenth, CLASS has not published an official fieldwork response rate for the 2023 individual file. We therefore report the public *N* = 11,670 and the complete-case sizes after listwise deletion (10,480 in the total-effect model; 10,093 in mediation). Post-stratification weights adjust for urban–rural residence, sex, and age, but cannot correct nonresponse related to unmeasured health or adversity; if missingness is related to poorer health or fewer social resources, complete-case estimates may be biased. Stereotype embodiment and aging attitudes may also shape how older adults report adaptation and health (34), a measurement issue that future multi-method designs could address.

Despite these limits, the study offers coherent evidence for aging policy. Childhood adversity remains relevant to late-life mental health in contemporary China, but its expression is concentrated in modifiable present-day conditions. Community-based strategies should combine adversity-informed screening, health management, adaptation-oriented psychosocial support, and meaningful digital engagement, an integrated agenda consistent with healthy aging frameworks and with emerging Chinese evidence on digital inclusion and social participation (1, 18, 21). Service intensity can be calibrated by subgroup risk, with priority attention to urban older adults with low community participation and stronger adversity linkage. Such an approach would align mental health prevention with the everyday settings in which older adults actually live, rather than treating depression as a problem visible only inside specialty clinics.

## Conclusion

This study shows that childhood adversity is associated with depressive symptoms in later life among Chinese older adults, and that this association is statistically accounted for predominantly by self-rated health, social adaptation, and internet media use in a parallel decomposition of cross-sectional data. Social adaptation and self-rated health showed the largest specific indirect associations and did not differ significantly from each other; digital participation provided a smaller additional association. These findings support integrated community interventions that address mental health as part of broader health management, social adaptation support, and digital inclusion rather than as an isolated clinical issue, while remaining explicit that the design cannot identify causal pathways. Targeted efforts for urban older adults with low community participation and more pronounced adversity exposure may offer a practical starting point for reducing preventable depressive burden in China’s aging society.

## Data Availability

Publicly available datasets were analyzed in this study. This data can be found here: http://class.ruc.edu.cn.
