# Supplementary material for: Associations between childhood adversity and depressive symptoms in later life: parallel mediation through self-rated health, internet media use, and social adaptation among Chinese older adults
Source: Front Public Health. 2026 Oct 8;14:1941289. doi: 10.3389/fpubh.2026.1941289 (PMC13686903; doi:10.3389/fpubh.2026.1941289)
Supplement: Supplementary file 1 [file Data_Sheet_1.PDF]

Supplementary Material

Associations Between Childhood Adversity and Depressive Symptoms in Later Life: Parallel Mediation Through Self-Rated Health, Internet Media Use, and Social Adaptation Among Chinese Older Adults

Supplementary Methods

Because Table 6 compares CES-D associations across subgroups, we tested whether the nine-item CLASS CES-D measures the same construct across sex, urban–rural residence, and age (60–74 versus 75 years or older). Items were treated as ordered categories (0–2 after reverse-coding of positive affect). The hypothesized structure is the conventional two-factor CES-D model: negative affect (items 2, 3, 5, 6, 7, and 8) and positive affect (items 1, 4, and 9). Complete-case Ns were 10,502 (sex and residence) and 10,480 (age).

All models used the weighted least squares mean- and variance-adjusted estimator (WLSMV) with theta parameterization in lavaan. Identification followed Wu and Estabrook (2016) via semTools::measEq.syntax (ID.fac = std.lv; ID.cat = Wu.Estabrook.2016). Configural models imposed no cross-group equality constraints. Metric invariance constrained thresholds and loadings. Scalar invariance constrained thresholds, loadings, and intercepts. Degrees of freedom were 52, 59, and 66, so each step is nested. Scaled CFI and RMSEA from the WLSMV chi-square are the decision indices. $\Delta CFI = CFI_{configural} - CFI_{scalar}$; $\Delta RMSEA = RMSEA_{scalar} - RMSEA_{configural}$. Conventional screens are $|\Delta CFI| < 0.010$ and $|\Delta RMSEA| < 0.015$ (Cheung & Rensvold, 2002; Chen, 2007). Nested chi-square tests used the Satorra (2000) scaled difference; with $N \approx 10{,}500$ they are reported but are not the invariance decision.

Supplementary Table 1. WLSMV fit of configural, metric, and scalar two-factor CES-D-9 models.

Grouping	Level	N	df	χ^2 scaled	CFI scaled	RMSEA scaled	SRMR
Sex	configural	10,502	52	569.790	0.9849	0.0436	0.0350
Sex	metric	10,502	59	550.426	0.9857	0.0398	0.0350
Sex	scalar	10,502	66	553.192	0.9858	0.0375	0.0351
Urban–rural	configural	10,502	52	638.668	0.9828	0.0464	0.0371
Urban–rural	metric	10,502	59	684.662	0.9817	0.0449	0.0379
Urban–rural	scalar	10,502	66	693.707	0.9816	0.0426	0.0380

Age (60–74 / 75+)	configural	10,480	52	579.851	0.9846	0.0440	0.0361
Age (60–74 / 75+)	metric	10,480	59	581.530	0.9848	0.0411	0.0365
Age (60–74 / 75+)	scalar	10,480	66	622.708	0.9838	0.0401	0.0366

Note. Estimator = WLSMV; parameterization = theta. CFI scaled and RMSEA scaled are based on the WLSMV scaled chi-square.

Supplementary Table 2. Configural-to-scalar change in fit and nested tests (WLSMV).

Grouping	N	Δdf	ΔCFI scaled	ΔRMSEA scaled	LRT p	Decision
Sex (male/female)	10,502	14	-0.0009	-0.0061	.173	Scalar invariance supported
Urban / rural	10,502	14	0.0012	-0.0038	< .001	Scalar invariance supported
Age 60–74 / 75+	10,480	14	0.0008	-0.0039	< .001	Scalar invariance supported

Note. ΔCFI = CFIconfigural − CFIscalar. ΔRMSEA = RMSEAscalar − RMSEAconfigural. Negative ΔCFI means CFI increased after constraints; negative ΔRMSEA means RMSEA decreased. Screens: |ΔCFI| < 0.010 and |ΔRMSEA| < 0.015. LRT is the 14-df Satorra (2000) scaled chi-square difference. Exact LRT p-values for residence and age were 2.48×10^{-12} and 4.01×10^{-9}. With N ≈ 10,500, significant chi-square tests detect trivially small discrepancies and do not override the ΔCFI / ΔRMSEA decision. Scalar invariance of the CES-D-9 is therefore supported, and subgroup comparisons of CES-D associations (Table 6 of the main text) are appropriate on measurement grounds.

Conclusion for subgroup analysis

Across sex, urban–rural residence, and age, scalar invariance holds by the conventional change-in-fit criteria. The CES-D-9 total score can be treated as comparable across these groups. Differences in the adversity–depression association in Table 6 may therefore be interpreted as heterogeneity in associations, not as an artifact of differential item functioning. Those subgroup contrasts remain descriptive; invariance justifies comparison, not a causal reading of the strata.
